# Joint Modeling of Multiple Social Networks to Elucidate Primate Social Dynamics: I. Maximum Entropy Principle and Network-Based Interactions

**DOI:** 10.1371/journal.pone.0051903

**Published:** 2013-02-28

**Authors:** Stephanie Chan, Hsieh Fushing, Brianne A. Beisner, Brenda McCowan

**Affiliations:** 1 Department of Statistics, University of California Davis, Davis, California, United States of America; 2 Department of Population Health & Reproduction, School of Veterinary Medicine, University of California Davis, Davis, California, United States of America; 3 Department of Anthropology, Pennsylvania State University, University Park, Pennsylvania, United States of America; 4 California National Primate Research Center, University of California Davis, Davis, California, United States of America; University of Namur, Belgium

## Abstract

In a complex behavioral system, such as an animal society, the dynamics of the system as a whole represent the synergistic interaction among multiple aspects of the society. We constructed multiple single-behavior social networks for the purpose of approximating from multiple aspects a single complex behavioral system of interest: rhesus macaque society. Instead of analyzing these networks individually, we describe a new method for jointly analyzing them in order to gain comprehensive understanding about the system dynamics as a whole. This method of jointly modeling multiple networks becomes valuable analytical tool for studying the complex nature of the interaction among multiple aspects of any system. Here we develop a bottom-up, iterative modeling approach based upon the maximum entropy principle. This principle is applied to a multi-dimensional link-based distributional framework, which is derived by jointly transforming the multiple directed behavioral social network data, for extracting patterns of synergistic inter-behavioral relationships. Using a rhesus macaque group as a model system, we jointly modeled and analyzed four different social behavioral networks at two different time points (one stable and one unstable) from a rhesus macaque group housed at the California National Primate Research Center (CNPRC). We report and discuss the inter-behavioral dynamics uncovered by our joint modeling approach with respect to social stability.

## Introduction

Networks are constructed in a wide variety of sciences, and these networks are popularly interpreted as an approximation of a complex system [1]. Network analyses, and computed patterns based on such networks, often provide valuable information or even uncover surprising patterns. Typical networks are based upon a single behavior, and are meant to approximate a single aspect of the study system. Hence the resultant analyses and patterns provide information for only one facet of the system of interest. However, dynamic systems, such as human and animal societies, usually consist of many facets working synergistically. Indeed, animal behaviorists construct multiple separate networks from a single target system [2], [3], [4]. Ideally, the information from these separate networks should be combined in order to achieve a comprehensive understanding of the system dynamics and processes. Unfortunately, the joint modeling methodologies and computational algorithms required to achieve a holistic understanding are still by and large missing.

In this paper we attempt to fill in this missing gap by proposing a maximum entropy principle based joint modeling methodology. The key features of this approach are that it is: 1) data-driven; 2) iterative; 3) and bottom-up. These features distinguish this modeling approach from most commonly - used modeling methods, which have a fixed and rigid model format, where all potential aspects of information are pre-selected. This old modeling perspective is particularly limited when our goal is to extract interaction relationships among multiple complex networks.

We illustrate and explain this new joint modeling approach using the rhesus macaque (*Macaca mulatta*) as an animal model. Four different social behaviors are considered: grooming, aggression, alliance, and status.

These four behaviors cover the major types of social interaction in rhesus macaques and constitute the main factors in understanding the social dynamics of the society as a whole. They are distinct networks that cover the same set of individual nodes. Many decades of research indicate that these behaviors are interrelated (e.g. both aggression and status are largely governed by dominance rank and are typically unidirectional), but joint modeling is required to quantify exactly how these behaviors co-vary. Furthermore, discovering what patterns of covariation are associated with stability vs. instability will improve our understanding of the evolution of sociality and grouping as well as improve captive management practices of socially-housed primates. We can use these patterns in developing and understanding the constraints over the overall network. The entropy can provide a measure of the structure compared to the independence among the four networks. Thus, rhesus macaques are a good model system in which to test joint modeling. The observed social behavior data come from observations on a captive group of rhesus macaques from the California National Primate Research Center (CNPRC). Our goal was to develop joint modeling techniques that allow the authentic and characteristic patterns of the system dynamics across these four social behavioral networks to be effectively summarized.

We construct four mono-behavioral social networks, including grooming, aggression, status and alliance derived from a single group (14B) with a fixed group membership (N = 77 monkeys). For simplicity of development as well as explanation, we use binary directed networks such that only the presence (or absence) and direction of a link between any pair of monkeys is considered. The frequency (i.e. weight) of an observed link between monkeys is ignored. In Part I this paper, we introduce this new joint modeling approach and its applications by focusing on coupling two behaviors (grooming and aggression). Then, in Part II we apply this joint modeling approach to all possible pair-wise combinations of the four behaviors and draw conclusions about its system dynamics. Joint modeling represents a significant improvement over standard social network analytical procedures, which are done in piecemeal fashion, extracting a single association between a response variable and a network measure from a single network. Joint modeling of two networks presents a new perspective on the synthesis of complex information, and sets the stage for extending this perspective to the synthesis of several interconnected networks.

## Methods

### Introduction to a maximum entropy based joint modeling approach

**Figure 1:**
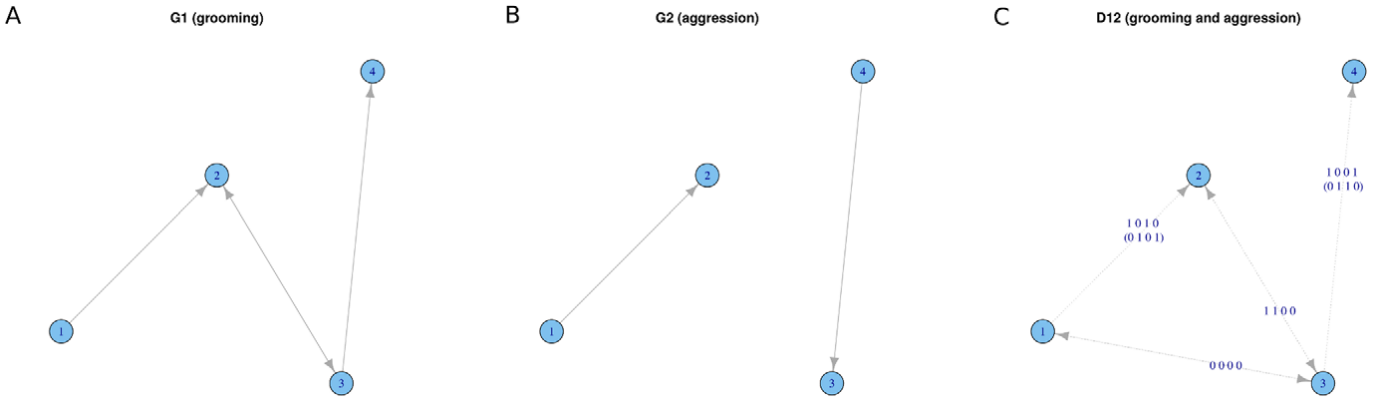
A visual example of jointly modeling two social networks: Groom and Aggression.

The basic idea of jointly modeling two binary (un-weighted) networks, corresponding to two types of social behaviors, is to prescribe the probability of a link in one network being associated a link in the other network. Each directed link is encoded as either 0 or 1, so a 2-dimensional binary code represents both directions of the relationship between two nodes. Therefore, the link between every pair of nodes in a two-behavior network is encoded by a 4-dimensional binary code. For example, let the two behaviors be grooming and aggression. A monkey dyad with mutual grooming, but no aggression can be represented by the 4-dimensional code vector 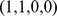 (see nodes 2, 3 in Figure 1). A pair of monkeys with opposite directional grooming and aggression is represented by a linkage vector 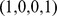 (see nodes 3,4 in Figure 1). Thus, there are 16 possible 4-dimensional linkage vectors, although there are only 10 biologically-distinct vectors. The empirical distribution of these 10 categories of linkage vectors represents the association information between these two behaviors of interest.

The application of our maximum entropy based joint modeling to such an empirical distribution then allows scientists to recreate a parametric probability distribution that explicitly embeds all key association information being revealed through the empirical distribution. The iterative steps are heuristically described as follows.

We begin with the distribution assuming independence between the two behaviors of interest (i.e. no association among the links). First, the four empirical marginal distributions of the vector codes are calculated and then the expected probabilities (or counts) of the 10 linkage vector categories are computed by assuming that each of the links of the two networks are independent. By comparing the empirical distribution with the expected one (assuming independence), any significant discrepancy in any category indicates a missing piece of information regarding the association between the two behaviors in the null model. It should be noted, however, that this is also subject to randomness of finite sampling.

Next, to correct any significant discrepancies we need to choose a constraint function that captures the missing association, and incorporate such a chosen constraint function into the revised version of probability distribution. The latter incorporation is the work of the maximum entropy principle, which chooses the maximum entropy one among all distributions fitting the constraints with empirical values calculated from the data. The advantage of using maximum entropy is that no extra or artificial assumptions are taken into the modeling. We now compare this new computed maximum entropy distribution with the empirical distribution; ideally the new distribution would include the right amount of association and improved in fitting the data.

We repeat this cycle of choosing a proper constraint function to describe the discrepancy and then updating the probability distribution until the discrepancy between the overall expected and empirical counts of the 10 categories is below a critical Chi-squared percentile.

We apply this joint modeling approach to real network data collected from one group (14B) at two different time points to perform three separate analyses: (1) using network data collected in 2009, when group 14B was regarded as a stable social group, and (2) using network data collected during a matched time period in 2011, prior to a social overthrow event in group 14B. (3) We also applied joint modeling to compare the behavior network in 2009 with 2011. In each analysis, the coupling of two behavioral networks reveals intrinsic and pertinent behavioral pattern knowledge in both years of data, respectively. Further the comparison of these two years analyses manifests critically important finding that provides a foundation for deriving monitoring measures for early warning signs of group instability and potential social overthrow.

### Ethics statement

All research reported in this manuscript adhered to the recommendations in the Guide for the Care and Use of Laboratory Animals of the National Institutes of Health, the laws of the United States government, and the recommendations of the Weatherall report, “The use of non-human primates in research.” All research subjects were housed in large social groups in half-acre outdoor enclosures to provide for their psychological wellbeing. The methodological approach was purely observational. All occurrences of illness or injury among study subjects were immediately reported to and treated by CNPRC veterinary staff, and all efforts were made to ameliorate suffering. This project was approved by the University of California, Davis Institutional Animal Care and Use Committee, protocol #11843.

### Binary behavioral network data

Consider four types of monkey behavioral interactions: grooming, aggression, alliance, and status. Grooming is defined as one monkey manipulating or picking through the fur of another using the fingers or lips. Aggression is defined as one monkey threatening, lunging at, chasing or biting another monkey, who typically responds with submission, such as moving away, running away, or screaming. An alliance is defined as a third party joining an on-going fight to help one of the original participants. Finally, status signaling is defined as a subordinate monkey giving a submission signal (silent-bared-teeth display, rump present, or move away) to a dominant animal in a peaceful context (i.e. aggression is not used to elicit submission). Each of these four behaviors is a directed action from one monkey to another. Thus, each action gives rise to a directed link in the social network.

**Figure 2:**
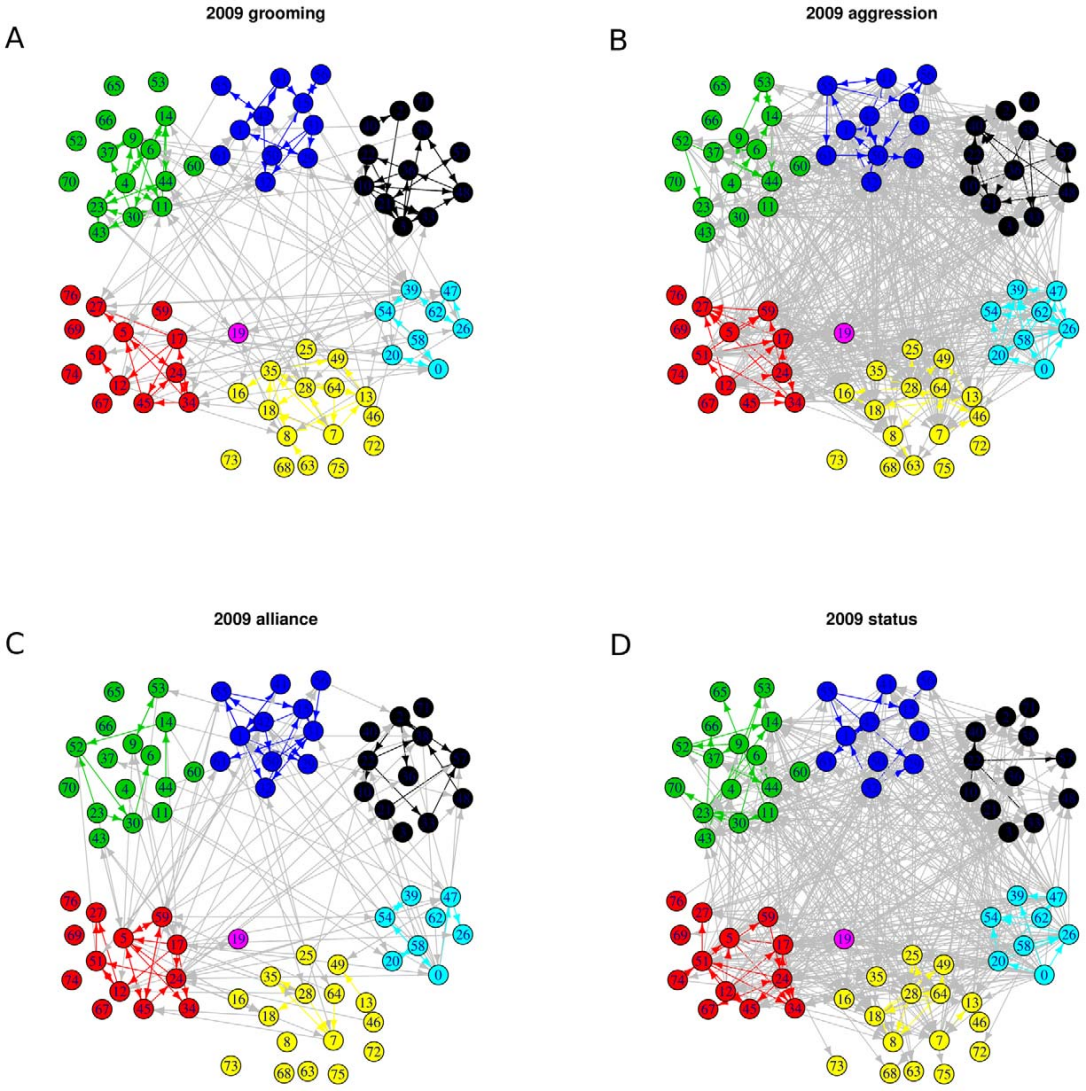
Empirical networks of the four behaviors for the study group (14B) during the stable time period in 2009.

We constructed four binary directed networks (i.e. ignoring the frequency/weight of each link) for each of the four behaviors, as show in Fig. 2, using behavioral data collected on group 14B at the CNPRC.

For each network, each node represents an individual monkey and an edge represents the directed relationship with an arrow going from the initiator to the recipient. The different colors represent the different matrilineal families in the group. Standard network analysis techniques focus on each network separately to try to extract behavioral specific pattern information. However, as animal behaviorists well know, these four networks are not independent of each other. For example, two allies may groom each other to help maintain their alliance relationship, and a subordinate who gives an unsolicited submission signal to a dominant is unlikely to also direct aggression towards that dominant animal [5]. These networks are thus bound together in multi-faceted and complex ways. In the next section we investigate how all pairs of the four behaviors are coupled together in order to discover their behavioral bonds. Through the coupling mechanisms via joint modeling we uncover informative structural information about these behavioral bonds.

### Maximum Entropy Modeling

The maximum entropy paradigm was introduced by physicist E.T. Jaynes [6], [7]. The ultimate goal of this principle is to determine the most likely probability distribution to minimize the number of structural assumptions while maximizing the amount of authentic information extracted from the observed data. This method is often used to describe network structures, as it has been successfully applied in a wide range of scientific fields such as physics [8], [9], neuroscience [10], [11], genetics [12], and computational linguistics [13]. The previous work uses the maximum entropy principle to model a specific network with one type of connection, but to our knowledge, we present the first application of the maximum entropy principle to the joint modeling of multiple networks over each type of connection. In the methodological description below, we lay out the fundamental basis of this application using rhesus macaque society as a model system.

Before formally developing our joint modeling framework, it is important to note that subject covariate information, such as gender, age or matriline membership are not used. Therefore we implicitly assume that the probability that one monkey interacts with any other monkey is equal across all pairs of monkeys. This assumption of equality of action is certainly not realistic, but it is a mean field approach commonly used in applications of statistical mechanics [10], [14], [15]. This mean field approach simplifies the maximum entropy computations. Additionally, it creates the null model to compare against the given data. This approximation will be updated in future work when including information from the individual covariates.

Let us generically represent an observed social behavior via a directed weighted network graph 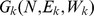 with 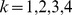 indicating four focal behaviors, where 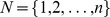 is the set of 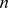 nodes (or subjects) of interest, the 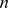-by-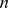 matrix 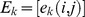 is the adjacency matrix of observed binary directed edges (or wiring), that is, 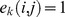 when there exists a directed link from node 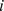 to node 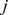, otherwise it is 0. The four matrices 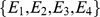 are graphically represented in the four panels of Fig. 1. 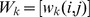 is the matrix of weights linking from node 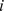 to node 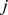.

For instance, in the two directed weighted network graphs [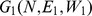,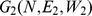] (grooming and aggression, respectively), we consider the simplest version of bivariate network relationship that is specified by the empirical distribution 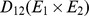 of the 4-dimensional binary linkage vectors, 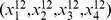. In the first column of Table 1, grooming is represented by the first two dimensions 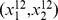 and aggression by the last two dimensions 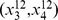. Observed counts of each linkage vector category are shown in the second column, and represent the empirical distribution 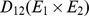, denoted by 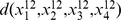. There can be a total of sixteen possible four-dimensional linkage vectors, and each edge could be represented in two ways: 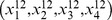 is equivalent to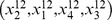. In the tables in this paper, we show only the 10 biologically-distinct 4-dimensional vectors and omit those that are repeated. The procedure of applying maximum entropy paradigm for modeling such an empirical distribution as given as follows:

**Table 1:** Maximum Entropy Calculations for Joint Modeling of Grooming and Aggression Networks.

grooming aggression	total	indep.	f1	f2	f3	f4
1 0 0 0	100	143.84 (13.36)	133.89 (8.58)	134.64 (8.91)	134.41 (8.81)	127.24 (5.83)
1 1 0 0	28	4.68 (116.04)	36.31 (1.90)	36.52 (1.99)	36.46 (1.96)	34.51 (1.23)
0 0 1 0	435	539.14 (20.12)	537.71 (19.62)	477.63 (3.81)	476.81 (3.67)	465.76 (2.03)
0 0 1 1	154	65.81 (118.17)	65.64 (118.96)	161.11 (0.31)	160.83 (0.29)	157.10 (0.06)
1 0 1 0	10	17.56 (3.25)	16.34 (2.46)	14.52 (1.41)	8.06 (0.47)	10.98 (0.09)
1 0 0 1	28	17.56 (6.21)	16.34 (8.31)	14.52 (12.52)	26.06 (0.14)	35.50 (1.59)
1 1 1 0	8	0.57 (96.49)	4.43 (2.87)	3.94 (4.19)	3.93 (4.21)	5.36 (1.31)
1 0 1 1	5	2.14 (3.81)	2.00 (4.53)	4.90 (0.00)	4.89 (0.00)	6.66 (0.41)
1 1 1 1	0	0.07 (0.07)	0.54 (0.54)	1.33 (1.33)	1.33 (1.33)	1.81 (1.81)
0 0 0 0	4575	4416.80 (5.67)	4405.07 (6.56)	4429.76 (4.76)	4422.09 (5.29)	4432.58 (4.58)
total 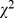		383.2001	174.3299	39.22938	26.16519	18.92789

The total column indicates the count for that type of edge. The numbers in each of the other columns indicate the expected number of edges under the distribution additionally including each constraint as well as the independent null distribution. The number in parenthesis is the Chi-squared value for that cell.

### Maximum entropy Procedure

#### Step-1

Find the expected counts for each specific link vector, denoted by 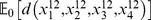, under the null model with independence among the four directed edges 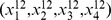. Then, compare these expected counts with the empirical count 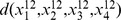, using the Chi-squared value 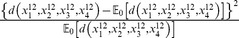 which is shown in parenthesis under the expected value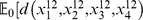. Look for a discrepancy to be addressed by step-2.

#### Step-2

We construct a function 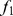 on the domain of 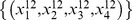 that is thought to repair the target discrepancy. So it is one important deviating direction of the null model from the underlying mechanism that generates the empirical distribution of 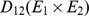. This function 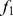 is chosen in such a way to satisfy the mean zero condition under the null distribution, that is, 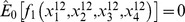, where 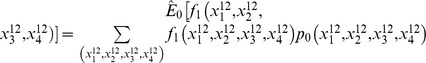. This condition means that this function 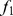
is perpendicular to the null model, meaning the features of 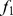 do not currently exist in the null model.

#### Step-3

We then accommodate the function 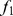 by modifying the null probability 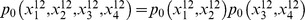 into a probability function 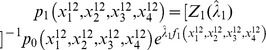 where the partition function 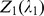 is the normalizing constant and 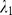 is chosen such that 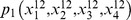 obtains the maximum entropy subject to the linear constraint 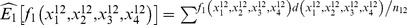 with total counts 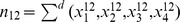.

#### Step-4

To compute the 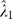, we need only to solve the following equation:
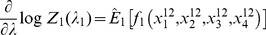
with the partition function 
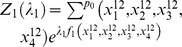
. This derivation is shown in more detail in Appendix S1. Then we check whether the total sum of Chi-squared values being smaller than the nominal critical Chi-squared percentile. If yes, we stop, otherwise we continue to the next step.

#### Step-5

We again compute the expected counts for all possible vector values of 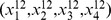 and find a target discrepancy to be repaired by the following performing steps 2 through 4 iteratively. Using 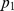, which we have now obtained, as the null probability, we repeat previous steps. First, choose a function 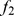 such that the expectation of 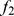 under the null 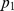 must be zero, 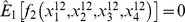. Then include the next function 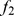 in the new probability by modifying the null probability into a probability function 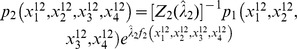 where the partition function 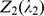 is the normalizing constant and 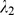 is determined from solving the maximum entropy with the empirical entropy linear constraint 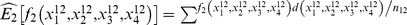. 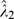 can be determined by repeating step 4.

This step is repeated until all observed counts and model-expected counts are closely matching to each other (not statistically significantly different). The decision can be made via Chi-squared testing with a suitable degree of freedom, that is, the total sum of Chi-squared values is smaller than the critical Chi-squared percentile.

Given that the 16 different 4-dimensional vectors represent only 10 biologically-distinct types of bivariate interaction, the appropriate degrees of freedom is 9 for the overall Chi-squared calculation, and the 95% and 99%-percentiles are 16.919 and 21.666, respectively. Thus, we stopped at the 4^th^ cycle of the above procedure when the total chi-squared is within the 99%-percentile in analysis on Table 1. Below we demonstrate how the maximum entropy works in an iterative fashion for all possible joint modeling for all bivariate behavioral networks.

## Results

### Joint modeling bivariate networks

The four directed binary networks via matrices 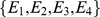 are rather sparse. The density, calculated by dividing the total number of observed edges by the total number of all possible edges in the graph) estimates the probability of observing an edge in each network over the three-month observation period in 2009. The probabilities of seeing a grooming edge is 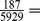
0.0315( = 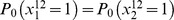), an aggression edge is 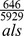
0.10896 ( = 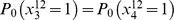), an alliance edge is 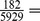
0.0263, and a status edge is 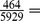
0.07826. Similar sparsity of edges is also seen over a corresponding three-month period in 2011.

### Further developments for finding function 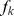


We further illustrate the development of this joint modeling approach using grooming and aggression networks as an example. We calculate our initial expected values assuming that aggression and grooming occur independently of one another. Thus, a high chi-squared value indicates that the observed frequency of each grooming-aggression combination is unlikely to be due to chance. A high chi-squared value also indicates a deviation between the null model and the underlying data-generating mechanism. Large discrepancies thus indicate the need to modify the null (independent) model.

We choose constraint functions that could modify the null model in a way such as to also cover the discrepancies between the expected counts and the observed counts. There are three considerations used when determining these functions. First, we use our subject knowledge to understand how the networks may be related, and then check how this relationship between networks can be demonstrated by looking at the differences between expected counts and observed counts. We can use this relationship to generate a constraint function, but create this function such that, under the null hypothesis, the expected value of the function is zero. This guarantees that the constraint model is not contained in the null model. The model created from the constraint functions is not necessarily a pre-determined model, rather this model is created from data observation and prior knowledge.

For example, there are two noticeably large chi-squared values for combinations 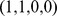 (bi-directional grooming) and 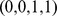 (bi-directional aggression). To separately capture these bi-directional grooming and aggression discrepancies between the expected and observed counts, we propose employ the two covariance-type of functions:
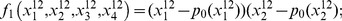


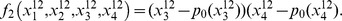
Via the above maximum entropy procedure for accommodating 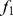 with the computed 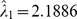, the expected value of 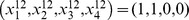 under first modified model is calculated as 36.31, which is much closer to the empirically observed count 28 than that of the null model 4.68. This is because the new model increases the probability of having bi-directional grooming. In the same way, when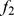 is also accommodated into the modified model with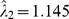, the discrepancy on 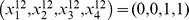 is drastically reduced from 118.96 to 0.31.

Even though significant discrepancies are reduced in two of the linkage vectors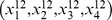, the remaining total Chi-squared values is still too high at 26.17. That is, there is still room for improving our modeling by accommodating more 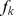
's. Notice that under the observed case, there are far more counts for 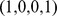 (28 observations) than for 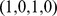 (10 observations). This means that there are 28 observed instances where monkey A grooms monkey B, but monkey B directs aggression at monkey A. This scenario is likely describes lower-ranking monkeys grooming higher-ranking monkeys as a way to pacify them after receiving aggression from the higher-ranking monkey or to discourage future aggression. Conversely, there are 10 instances in which monkey A both grooms and directs aggression at monkey B. This scenario likely represents a dominant animal initiating both grooming and aggression toward a subordinate.

To modify our joint model for accommodating this fact, we propose a third function 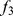 as follows:
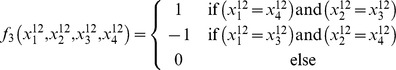
The idea behind this functional construction are the facts that 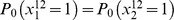 and 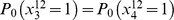. By such a design, this constraint function increases the probability when the two directions are opposite and decreases the probability by the same amount when the two directions face the same way. After applying maximum entropy procedure with calculated 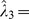
0.586, the total chi-squared value decrease again. However it is still too high.

To further improve our modeling for fitting the data better, we identify all vectors values of 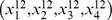, except the two cases 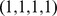 and for 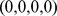, when compared to the expected number of counts, that tends to have more empirical counts than expected ones where both some grooming (or the first network type) and some aggression (or the second network type) occurs rather than only grooming or only aggression. We quantify this observation by choosing the following function
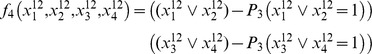
where 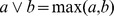. This function indeed helps our modeling by reducing the overall discrepancy under the critical value of 1% nominal level with 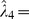
0.39

**Figure 3:**
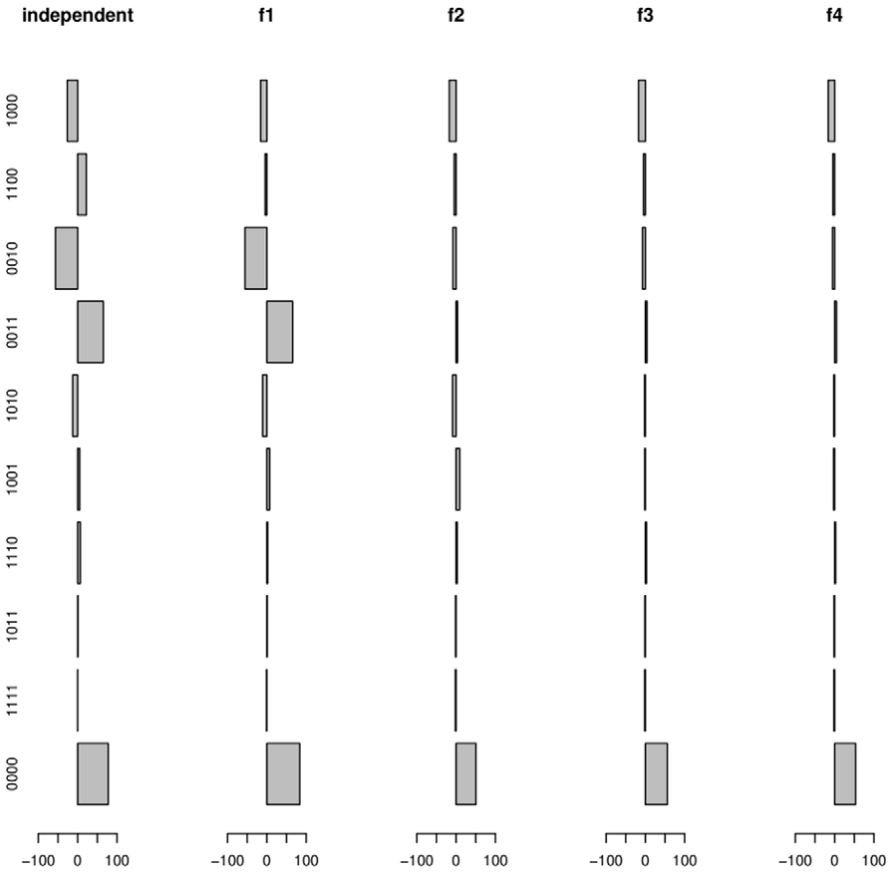
Histograms of the expected frequency of each linkage vector category under the null model of independence and after the cumulative application of the four constraint functions.

In Figure 3, we can see the differences between the computed expected number for each type of link and the observed number. The expected distribution is under the assumption that each link is independent for the rest, which is clearly not true. The first function (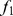) adjusts for the covariance association in the first network (grooming), and this adjustment is most apparent in the first two linkage vectors 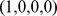
and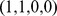, as these bars approach zero. The second function (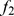) adjusts for the covariance in the second network (aggression), and this is most evident in the two linkage vectors corresponding to aggression 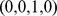
and 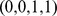, as these bars approach zero. These covariance adjustments generate an expected probability that is closer to the observed value. The third function (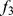) adjusts for the opposite directions between 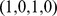
and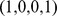, in order to pull these discrepancies toward zero. This can be seen as the fifth and sixth bars approach zero in 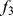. Looking at the differences we still see that the first four bars (where only one of the networks has an occurrence) have lower observed than expected values, and the last six bars (where either both or neither networks have an occurrence) have higher observed than expected value. The fourth function (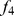) aims to adjust for the covariance between the two networks, which decreases the expected value for the first four vectors and increases the expected value for the other six vectors. This function brings all of the discrepancies closer to zero. There appear to be more links of 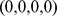
than expected over all four functions. However, the Chi-squared value for 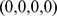 is considerably low, because the difference is low compared to the relatively high number (4575) of non-connected links.

### Application of joint modeling to all pair-wise networks

Through this development of bottom-up maximum entropy paradigm based joint modeling, we see how this data-driven procedure helps our understanding of the interaction between grooming and aggression. In the same way, we can gain an understanding for all other pair-wise behaviors by deriving their specific set of 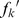
s. Heuristically these sets of functions might be very similar or very different.

**Figure 4:**
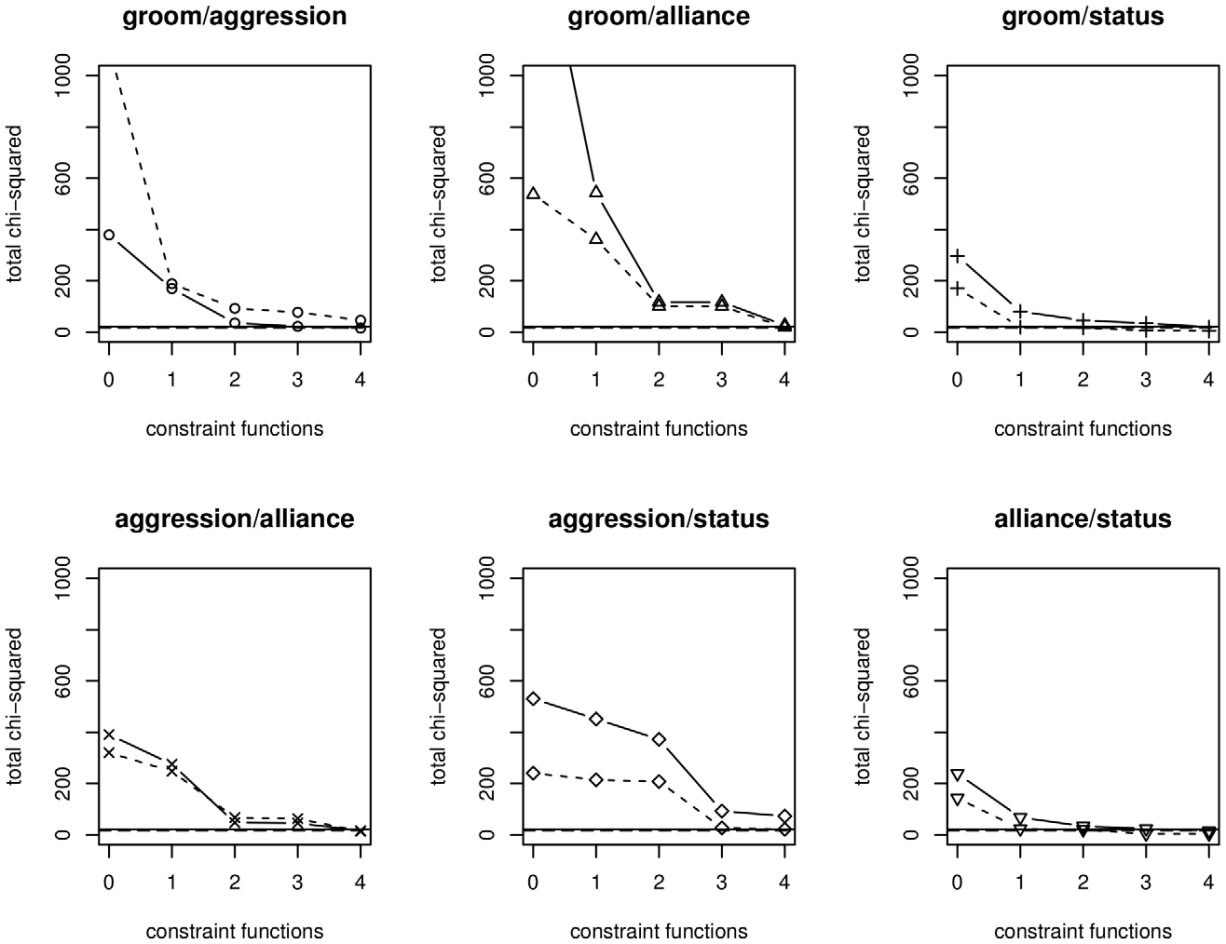
Plots of the change in total chi-squared value after the cumulative application of the four constraint functions for all bivariate networks.

Below we check whether the set of four functions 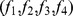, derived from exploring the interacting relationship between grooming and aggression, retain universal effects through all other pairwise behaviors on 2009 data and across data of different year 2011. We report the series of tables of our joint modeling analyses (see Tables 2 and 3, as well as Tables S1, S2, S3, S4, S5, S6, S7, S8, S9, S10, S11, S12). Figure 4 shows the decreasing Chi-squared values as the number of constraining functions increase, also see Tables 4 and 5. This means that as we increase more functions to describe associations, the expected distribution more closely approximates the observed distribution. These plots also show that for the most part, the 2011 data fits better than the 2009 data, so there may be less complex associations in 2011 just before the social overthrow.

**Table 2:** Total chi-squared values of iterative joint modeling on 2009.

2009	indep	f1	f2	f3	f4
groom/aggression	383.2001	174.3299	39.22938	26.16519	18.92789
groom/alliance	1746.551	545.8951	120.1105	119.9372	29.34931
groom/status	297.0714	81.04574	49.71641	38.7586	23.72612
aggression/alliance	395.317	279.2095	51.46468	49.42425	16.10065
aggression/status	537.9403	458.3071	383.1573	106.3561	84.88729
alliance/status	238.1352	67.86995	36.82985	27.28788	18.85823

**Table 3:** Total chi-squared values of iterative joint modeling on 2011.

2011	indep	f1	f2	f3	f4
groom/aggression	1115.901	190.8692	93.95191	78.73596	47.23078
groom/alliance	538.8094	363.3119	103.3141	102.7869	21.09991
groom/status	170.4748	19.56063	16.78067	6.9537	4.810583
aggression/alliance	323.3224	250.005	69.68484	65.91343	13.6323
aggression/status	243.5242	217.1091	210.8734	31.75803	25.63412
alliance/status	142.4655	23.5324	20.59748	4.070777	3.872492

**Table 4:** Total Chi-squared values for iterative joint modeling between 2009 and 2011.

2009/2011	indep	f1	f2	f3	f4
groom	387.589	99.92067	51.10631	50.92136	11.00127
aggression	107.952	93.91709	94.26985	21.58309	21.43661
alliance	156.1359	88.60414	49.01379	48.23746	8.40272
status	93.48194	64.61319	56.21222	12.63886	12.818

**Table 5:** 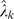
's values of iterative joint modeling between 2009 and 2011.

2009/2011	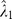	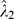	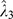	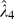
groom	1.743028	1.132691	−0.1235996	1.232784
aggression	0.6215508	0.05868105	−0.9726387	0.01167172
alliance	1.731542	1.46947	−0.2765222	1.396532
status	−2.193231	−0.9594662	−0.9828907	−0.06636444

We also performed the joint modeling over the 2009/2011 data for each behavior. In these cases, we only used the individuals who appeared in both the 2009 data set and 2011 data set for each behavior. These results are given in Tables 4 and 5 (and Tables S13, S14, S15, S16).

## Discussion

Joint modeling involves the empirical construction of multiple social networks to synthesize complex information and reveal collective behaviors which arise from several interconnected social realms. Our approach recreates the joint probabilities of two types of social relationships, such as grooming and aggression, by first using the raw data to calculate expected probabilities of jointly observing grooming and aggression for a given dyad, assuming grooming and aggression relationships are independent. Constraint functions are then iteratively applied to tune these probabilities to match the observed network data.

We jointly modeled all bivariate behavioral networks, using four constraint functions 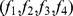, to investigate the inter-behavioral relationships among four social networks in a rhesus macaque society at two different time points: 2009 and 2011. The study system was stable throughout 2009, but became unstable in 2011, which culminated in a social collapse in Fall 2011. This society, therefore, represents an ideal system in which to test these new joint modeling techniques because the inter-behavioral dynamics of the system changed from 2009 to 2011, which allows the identification of meaningful inter-behavioral dynamics associated with system instability. Below we discuss the results of our joint modeling efforts with respect to the change in social stability of the study system.

### Inter-behavioral patterns: hallmarks of rhesus society

First, we note that our joint modeling approach confirmed several known behavioral patterns in rhesus macaque society. For example, aggression and status interactions are governed by dominance, and as such, the direction of these interactions is highly predictable [16]. Indeed, rhesus macaques are highly despotic, showing a high degree of asymmetry in their aggressive interactions [17]. Conversely, grooming and alliance interactions occur in multiple contexts, thus the direction of these interactions is less predictable outside of the context of kinship, sex, age, and rank. For instance, a subordinate may groom a dominant to gain tolerance at a feeding site, or a dominant may groom a subordinate after a fight to reconcile [18], [19]. Both known patterns emerge in the joint modeling. In the Aggression & Status joint networks, same direction status and aggression observed more frequently than expected by chance and were far more common than opposite direction status and aggression (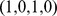 vs. 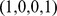: 147 vs. 2 in 2009 and 78 vs. 3 in 2011 See Tables S3 and S9). In the Grooming & Alliance joint networks, same direction groom and alliance were just as common as opposite direction groom and alliance (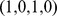 vs. 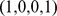: 9 vs. 10 in 2009; 13 vs. 11 in 2011; see Tables S2 and S8).

Joint modeling over 2009 and 2011 further demonstrate the above patterns. For both Aggression 2009–2011 and Status 2009–2011 networks, the third constraint function described the expected distribution quite well, as the overall chi-squared value decreased significantly with f3 (see Table 4). The third function checks for opposite direction links (i.e. change in the direction of aggression from 2009 to 2011), and the negative values of 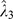 revealed that very few dyads changed the direction of aggression or status from 2009 to 2011. 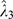 For the Groom and Alliance 2009–2011 networks, the fourth constraint function described the expected distribution well as the overall chi-squared value showed a large decrease, while the third function had little effect. The direction of groom and alliance interactions is more variable because both behaviors can be directed from subordinate to dominant or vice versa, depending upon the social context. Therefore, the fourth function, which checked for covariance between 2009 and 2011 for groom and alliance, revealed that it was more likely for a link to occur in both 2009 and 2011 than to occur in only one of the networks (note high 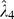 values in Table 5).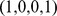 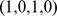 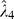. Among the individuals who appeared in both the 2009 and 2011 networks, the direction of behavior was not fixed for grooming and alliance behaviors.

### Inter-behavioral patterns of instability

#### Unwilling to show status

The results of several different bivariate behavioral networks indicate confusion and upheaval in the social dynamics of dominance. The number of edges stayed relatively the same from 2009 to 2011 for grooming (187 vs. 168), alliance (182 vs. 202), and aggression (646 vs. 549), but decreased by nearly half for status, from 464 to 266. Using a Pearson chi-squared test for the contingency of the edges for 2009 and 2011, we find a test-statistic of 31.57, with three degrees of freedom, this has a p-value of 5.43×10^−7^. When comparing the proportions of the individual types of edges using the Pearson chi-squared test, grooming and aggression return test statistics 1.21 and 1.76 respectively, which do not indicate a change. The proportion of alliance edges increased from 0.123 to 0.170 with test-statistic of 11.60 and p-value 6.58×10^−4^. The proportion of status edges decreased from 0.314 to 0.224 with test statistic 25.90 and p-value 3.60×10^−7^. We discuss the decrease in status edges. Essentially, animals were less willing to peacefully communicate their dominance relationships in 2011 than they had been in 2009. This finding is consistent with our previous work which indicates that status signals, especially silent-bared-teeth displays (SBTs), contribute to group stability. For example, groups require a sufficient number of policers to mitigate conflicts among group members [20], [21] and these key individuals acquire their role as policers by receiving peaceful status signals from group members [22]. A decrease in willingness to communicate status may result in an insufficient number of individuals with high enough social power to police the group, thereby allowing outbreaks of deleterious aggression. The joint modeling reveals additional details about the trend of unwillingness to show status.

Second, the Aggression & Status bivariate networks (Tables S3 and S9) show a change in the nature of the relationship between these behaviors from 2009 to 2011, which point to upheaval in the dominance hierarchy. We found a 50% decrease from 2009 to 2011 in two linkage vectors: one-way status without aggression 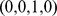
aggression and status in the same direction 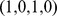 (289 vs. 172 and 147 vs. 78, respectively. The chi-squared test-statistics for this decrease is 51.17 and 22.77 with degree of freedom one, thus showing statistical significance). Given that counts of one-way aggression without status are similar in both years, it appears that subordinates were less willing to give status signals to dominants in 2011 than in 2009, regardless of their aggressive interactions. We also saw a change in the ratio of one-way aggression without status 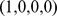 to one-way status without aggression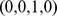 from 1∶1.15 in 2009 to 1∶2.16 in 2011. (This has statistical significance with a test-statistic of 26.38 and p-value 3.61×10^−7^.) Since status signals are peaceful expressions of subordination [5] given to avoid aggression, it is not surprising that the stable time period shows relatively more one-way status without aggression.

Third, patterns of bi-directional aggression with respect to status also indicate instability in dominance relationships. The proportion of bi-directional aggression dyads that also include status decreased by 50% (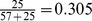 in 2009; 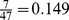 in 2011. The test-statistic of this decrease is 3.10 with p-value of 0.078). This indicates that in 2009, many dyads with bi-directional aggression still had well-established dominance relationships, as evidenced by one-way status. However, fewer such dyads existed in 2011, suggesting that little bi-directional aggression occurred between animals with clear, settled ranks. In a stable social system, subordinates occasionally protest the actions of a dominant animal (protecting offspring or defending important resources) without posing a threat to the dominant's social standing [17]. These data suggest that during 2011 subordinates may have been unable to safely protest aggression by dominants without appearing to threaten the dominant's social rank and that the bi-directional aggression observed may have been of a more serious nature, constituting rank challenges against dominant animals rather than temporary protests.

Finally, unusual bi-directional status interactions were observed in 2011 that were absent in 2009. Status signals normally unidirectional and occur in dyads with well-established dominance relationships, because the subordinate anticipates losing an impending contest with the dominant, and submits before a fight ensues [23]. Bi-directional status suggests a switch in dominance over the course of the observation period. However, four of the six counts occurred in dyads with one-way aggression with bi-directional status, suggesting that no (or very little) fighting occurred in the process of this switch, which further implies a rapid switch in dominance, rather than a drawn out fight between the two combatants. Regardless, unusual mutual status is consistent with the general finding that dominance relationships were in a state of upheaval in 2011.

#### Unusual coupled mutual behaviors

In 2009, we observed coupled mutual behavior 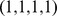 in the Grooming & Alliance bivariate network *only* – animals groomed and offered alliance support in both directions (Table S3). However, in 2011, we observed non-zero 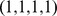 counts in two antagonistic behaviors: (1) Grooming & Aggression, and (2) Aggression & Alliance, while the mutual cohesive behavior seen in 2009 for Grooming & Alliance bivariate network is absent (Tables S6, S8, S10). As mutual grooming and alliance interactions are positive, cohesive behaviors in which direction can go both ways, their presence is expected in a stable social system. Absence of mutual grooming and alliance support in 2011 may suggest a breakdown of the cohesive elements of the society. However, mutual grooming and aggression presents a confusing combination, as does mutual alliance and aggression, because the bi-directionality in these behaviors is in opposition. Bi-directionality is cohesive in grooming and alliance networks, but is antagonistic and divisive in aggression network. These dyads seem to be potential allies who are struggling between grooming to maintain coalitionary ties and taking advantage of the social opportunity to challenge one another's rank. Two of the six dyads are kin, and this may be an indicator of instability, as cohesive kin relationships are crucial to group stability [3]. Mutual aggression and grooming/alliance in non-kin dyads may also signal instability, especially if those dyads represent critical alliances for the maintenance of rank.

#### Loss of social complexity

Finally, the bivariate behavioral networks from 2011 are fit much better by the four constraint functions than those from 2009, with the exception of the groom-aggression network in 2009, from which the four constraint functions were originally derived, see Table 2 and 3. One of the key reasons is the observation of mutual status in 2011, but not in 2009. From a modeling perspective, this non-zero count fits better to the null model of independence, indicating that the model structure is much simpler. However, from a biological perspective, the behavioral dynamics of the group seem to lose important structural subtlety in 2011 that is normally found in a stable system.

Joint modeling of both Grooming & Status and Alliance & Status in 2011 also shows a loss of structural complexity. Both sets of bivariate networks do not need to go into the stages of accommodating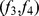. These two bivariate behaviors are particularly well modeled by only accommodating 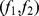 into the independence null model, indicating that the association between behaviors is simpler in structure in 2011 than in 2009. Thus, much of the covariance between Grooming & Status and Alliance & Status networks is lost in 2011. We speculate this loss of structural complexity in behavioral dynamics is associated with the social instability in the monkey's society, as implied by the social collapse observed later that year. The precise nature of this reduced social complexity will be explored in future papers.

## Conclusions

Joint modeling of multiple networks is essential to gain a more realistic understanding of social dynamics because many global patterns, such as those in health, social stability, and social hierarchies, arise from multiple interconnected networks. The piecemeal approach of standard social network analysis is insufficient for providing the complex information required to realistically and holistically assess and extract dynamic and causal processes involved in the emergence of collective behavior. We developed a bottom-up, iterative modeling approach based upon the maximum entropy principle, deriving multiple constraint functions to approximate the bivariate relationship between two jointly modeled networks. Our results not only confirm known patterns of social behavior in rhesus macaques, but also identify new inter-behavioral dynamics associated with social instability, including significant changes in the nature of the bivariate relationship between Aggression & Status which reflect unsettled dominance, the appearance of unusual (antagonistic) coupled mutual behaviors (e.g, Groom & Aggression), and decreased inter-behavioral complexity in Groom & Status and Alliance & Status networks.

Joint modeling may be done horizontally over multiple networks at the same scale, as we have shown here, or vertically across multiple scales, such as the genetic level, the individual level, and the family or community level. Information from individual characteristics may also be used to classify and model the structure. Our future work intends to include the sex and the matriline network information in the joint modeling. Joint modeling over networks can have a wide application in multiple fields. We can use joint modeling to understand the various levels of any social network such as friendship, work partnership, or location proximity as long as the networks are defined by similarity in the nodes (the same individuals, businesses, institutions, species, etc.) in the data set. For example, to better understand the emergence of health outcomes such as disease joint modeling can determine how direction of disease transfer overlaps with family relations.

In economics, joint modeling may be used to combine information from import/export of various commodities where each commodity may be a different level. Airline and road networks may be compared over individual cities. The joint modeling approach using maximum entropy can determine which type of connection and correlation may exist across networks. In sum, the joint modeling approach described here will facilitate the detection of emergent global patterns in a wide variety of disciplines, ranging from behavior biology, ecology, genetics, and epidemiology to economics and transportation.

## Supporting Information

Table S1 Maximum entropy calculations for joint modeling of Grooming and Status networks in 2009. The total column indicates the total observed count for that type of edge. The numbers in each of the other columns indicate the expected number of edges under the distribution additionally including each constraint as well as the independent null distribution. The number in parentheses is the Chi-squared value for that cell.
(DOCX)

Table S2 Maximum entropy calculations for joint modeling of Grooming and Alliance networks in 2009. The total column indicates the total observed count for that type of edge. The numbers in each of the other columns indicate the expected number of edges under the distribution additionally including each constraint as well as the independent null distribution. The number in parentheses is the Chi-squared value for that cell.
(DOCX)

Table S3 Maximum entropy calculations for joint modeling of Aggression and Status networks in 2009. The total column indicates the total observed count for that type of edge. The numbers in each of the other columns indicate the expected number of edges under the distribution additionally including each constraint as well as the independent null distribution. The number in parentheses is the Chi-squared value for that cell.
(DOCX)

Table S4 Maximum entropy calculations for joint modeling of Aggression and Alliance networks in 2009. The total column indicates the total observed count for that type of edge. The numbers in each of the other columns indicate the expected number of edges under the distribution additionally including each constraint as well as the independent null distribution. The number in parentheses is the Chi-squared value for that cell.
(DOCX)

Table S5 Maximum entropy calculations for joint modeling of Alliance and Status networks in 2009. The total column indicates the total observed count for that type of edge. The numbers in each of the other columns indicate the expected number of edges under the distribution additionally including each constraint as well as the independent null distribution. The number in parentheses is the Chi-squared value for that cell.
(DOCX)

Table S6 Maximum entropy calculations for joint modeling of Grooming and Aggression networks in 2011. The total column indicates the total observed count for that type of edge. The numbers in each of the other columns indicate the expected number of edges under the distribution additionally including each constraint as well as the independent null distribution. The number in parentheses is the Chi-squared value for that cell.
(DOCX)

Table S7 Maximum entropy calculations for joint modeling of Grooming and Status networks in 2011. The total column indicates the total observed count for that type of edge. The numbers in each of the other columns indicate the expected number of edges under the distribution additionally including each constraint as well as the independent null distribution. The number in parentheses is the Chi-squared value for that cell.
(DOCX)

Table S8 Maximum entropy calculations for joint modeling of Grooming and Alliance networks in 2011. The total column indicates the total observed count for that type of edge. The numbers in each of the other columns indicate the expected number of edges under the distribution additionally including each constraint as well as the independent null distribution. The number in parentheses is the Chi-squared value for that cell.
(DOCX)

Table S9 Maximum entropy calculations for joint modeling of Aggression and Status networks in 2011. The total column indicates the total observed count for that type of edge. The numbers in each of the other columns indicate the expected number of edges under the distribution additionally including each constraint as well as the independent null distribution. The number in parentheses is the Chi-squared value for that cell.
(DOCX)

Table S10 Maximum entropy calculations for joint modeling of Aggression and Alliance networks in 2011. The total column indicates the total observed count for that type of edge. The numbers in each of the other columns indicate the expected number of edges under the distribution additionally including each constraint as well as the independent null distribution. The number in parentheses is the Chi-squared value for that cell.
(DOCX)

Table S11 Maximum entropy calculations for joint modeling of Alliance and Status networks in 2011. The total column indicates the total observed count for that type of edge. The numbers in each of the other columns indicate the expected number of edges under the distribution additionally including each constraint as well as the independent null distribution. The number in parentheses is the Chi-squared value for that cell.
(DOCX)

Table S12

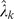
 values of iterative joint modeling on 2009 and 2011.(DOCX)

Table S13 Maximum entropy calculations for joint modeling of Grooming networks from 2009 and 2011. The total column indicates the total observed count for that type of edge. The numbers in each of the other columns indicate the expected number of edges under the distribution additionally including each constraint as well as the independent null distribution. The number in parentheses is the Chi-squared value for that cell.
(DOCX)

Table S14 Maximum entropy calculations for joint modeling of Aggression networks from 2009 and 2011. The total column indicates the total observed count for that type of edge. The numbers in each of the other columns indicate the expected number of edges under the distribution additionally including each constraint as well as the independent null distribution. The number in parentheses is the Chi-squared value for that cell.
(DOCX)

Table S15 Maximum entropy calculations for joint modeling of Alliance networks from 2009 and 2011. The total column indicates the total observed count for that type of edge. The numbers in each of the other columns indicate the expected number of edges under the distribution additionally including each constraint as well as the independent null distribution. The number in parentheses is the Chi-squared value for that cell.
(DOCX)

Table S16 Maximum entropy calculations for joint modeling of Status networks from 2009 and 2011. The total column indicates the total observed count for that type of edge. The numbers in each of the other columns indicate the expected number of edges under the distribution additionally including each constraint as well as the independent null distribution. The number in parentheses is the Chi-squared value for that cell.
(DOCX)

Appendix S1 (DOCX)

## References

[1] Barabasi AL (2009) Scale-free networks: a decade and beyond. Science 325: 412–413. 19628854 10.1126/science.1173299

[2] Flack JC, Girvan M, de Waal FBM, Krakauer DC (2006) Policing stabilizes construction of social niches in primates. Nature 439: 426–429. 16437106 10.1038/nature04326

[3] Beisner BA, Jackson ME, Cameron A, McCowan B (2011) Detecting instability in animal social networks: genetic fragmentation is associated with social instability in rhesus macaques. PLoS ONE 6: e16365. 21298105 10.1371/journal.pone.0016365 PMC3027651

[4] Beisner BA, Jackson ME, Cameron A, McCowan B (2011) Effects of natal male alliances on aggression and power dynamics in rhesus macaques. American Journal of Primatology 73: 790–801. 21698659 10.1002/ajp.20907 PMC3058123

[5] Flack JC, de Waal FBM (2007) Context modulates signal meaning in primate communication. Proceedings of the National Academy of Science 104: 1581–1586. 10.1073/pnas.0603565104 PMC1780279 17244712

[6] Jaynes ET (1957) Information theory and statistical mechanics. Physical Review 108: 171–190.

[7] Rosenkrantz RD, editor (1983) E. T. Jaynes: Papers on probability, statistics, and statistical physics. Dordrecht: Kluwer Academic Publishers.

[8] Bialek W, Ranganathan R (2007) Rediscovering the power of pairwise interactions. arXiv:07124397v1 [q-bioQM]: Cornell University Library.

[9] Stephens GJ, Bialek W (2010) Statistical mechanics of letters in words. Physical Review E 81: 066119. 10.1103/PhysRevE.81.066119 PMC3648583 20866490

[10] Schlens J, Field GD, Gauthier JL, Grivich MI, Petrusca D, et al (2006) The structure of multi-neuron firing patterns in primate retina. Journal of Neuroscience 26: 8254–8266. 16899720 10.1523/JNEUROSCI.1282-06.2006 PMC6673811

[11] Tang A, Jackson D, Hobbs J, Chen W, Smith JL, et al (2008) A maximum entropy model applied to spatial and temporal correlations from cortical networks in vitro. Journal of Neuroscience 28: 505–518. 18184793 10.1523/JNEUROSCI.3359-07.2008 PMC6670549

[12] Lezon TR, Banavar JR, Cieplak M, Maritan A, Fedoroff NV (2006) Using the principle of entropy maximization to infer genetic interaction networks from gene expression patterns. Proceedings of the National Academy of Science 103: 19033–19038. 10.1073/pnas.0609152103 PMC1748172 17138668

[13] Berger AL, Della Pietra SA, Della Pietra VJ (1996) A maximum entropy approach to natural language processing. Computational Linguistics 22: 39–71.

[14] Tkacik G, Schneidman E, Berry MJ, Bialek W (2009) Spin glass models for a network of real neurons. arXiv:09125409 [q–bioNC]: Cornell University Library.

[15] Schneidman E, Still S, Berry MJ, Bialek W (2003) Network information and connected correlations. Physical Review Letters 91: 238701. 14683220 10.1103/PhysRevLett.91.238701

[16] Sade DS (1969) An algorithm for dominance relations: Rules for adult females and sisters. American Journal of Physical Anthropology 31: 271.

[17] Thierry B (2004) Social epigenesis. In: Thierry B, Singh M, Kaumanns W, editors. Macaque Societies. Cambridge: Cambridge University Press. pp. 267–290.

[18] Aureli F, Cords M, van Schaik CP (2002) Conflict resolution following aggression in gregarious animals: a predictive framework. Animal Behaviour 64: 325–343.

[19] Barrett L, Gaynor D, Henzi SP (2002) A dynamic interaction between aggression and grooming among female chacma baboons. Animal Behaviour 63: 1047–1053.

[20] Beisner BA, Jackson ME, Cameron A, McCowan B (2012) Sex ratio, conflict dynamics and wounding in rhesus macaques (*Macaca mulatta*). Applied Animal Behaviour Science 137: 137–147. 22628902 10.1016/j.applanim.2011.07.008 PMC3357203

[21] McCowan B, Beisner BA, Capitanio JP, Jackson ME, Cameron A, et al (2011) Network stability is a balancing act of personality, power, and conflict dynamics in rhesus macaque societies. PLoS ONE 6: e22350. 21857922 10.1371/journal.pone.0022350 PMC3153932

[22] Beisner BA, Jackson ME, Seil S, Atwill ER, McCowan B (submitted) Reputation and intent of key policers underlies network stability in rhesus macaque societies. Proceedings of the Royal Society B

[23] de Waal FBM, Luttrell LM (1985) The formal hierarchy of rhesus macaques: an investigation of the bared-teeth display. American Journal of Primatology 9: 73–85. 10.1002/ajp.1350090202 32102494

