# Supplementary material for: Joint Modeling of Multiple Social Networks to Elucidate Primate Social Dynamics: I. Maximum Entropy Principle and Network-Based Interactions
Source: PLoS One. 2013 Feb 28;8(2):e51903. doi: 10.1371/journal.pone.0051903 (PMC3585323; doi:10.1371/journal.pone.0051903)
Supplement: Table S2 — Maximum entropy calculations for joint modeling of Grooming and Alliance networks in 2009. The total column indicates the total observed count for that type of edge. The numbers in each of the other columns indicate the expected number of edges under the distribution additionally including each constraint as well as the independent null distribution. The number in parentheses is the Chi-squared value for that cell. (DOCX) [file pone.0051903.s002.docx]

Table S2 Maximum entropy calculations for joint modeling of Grooming and Alliance networks in 2009

| grooming  alliance | total | indep | f1 | f2 | f3 | f4 |
| --- | --- | --- | --- | --- | --- | --- |
| 1 0 0 0 | 118 | 171.70 (16.79) | 159.82 (10.94) | 159.50 (10.80) | 159.50 (10.80) | 145.09 (5.06) |
| 1 1 0 0 | 28 | 5.59 (89.80) | 43.35 (5.43) | 43.26 (5.38) | 43.26 (5.38) | 39.35 (3.28) |
| 0 0 1 0 | 98 | 142.46 (13.88) | 142.09 (13.68) | 133.81 (9.58) | 133.81 (9.58) | 121.75 (4.63) |
| 0 0 1 1 | 18 | 3.85 (52.01) | 3.84 (52.23) | 30.92 (5.40) | 30.92 (5.40) | 28.14 (3.65) |
| 1 0 1 0 | 9 | 4.64  (4.10) | 4.32  (5.07) | 4.07  (5.98) | 3.60  (8.11) | 13.04 (1.25) |
| 1 0 0 1 | 10 | 4.64  (6.19) | 4.32  (7.47) | 4.07  (8.65) | 4.60  (6.35) | 16.67 (2.67) |
| 1 1 1 0 | 7 | 0.15 (310.44) | 1.17 (29.00) | 1.10 (31.52) | 1.10 (31.52) | 4.00  (2.25) |
| 1 0 1 1 | 6 | 0.13 (275.26) | 0.12 (296.60) | 0.94 (27.24) | 0.94 (27.24) | 3.41  (1.97) |
| 1 1 1 1 | 2 | 0.00 (975.66) | 0.03 (122.40) | 0.25 (11.95) | 0.25 (11.95) | 0.92  (1.25) |
| 0 0 0 0 | 5385 | 5272.12 (2.42) | 5258.12 (3.06) | 5247.58 (3.60) | 5247.48 (3.60) | 5252.68 (3.33) |
| total $\chi^{2}$ |  | 1746.551 | 545.8951 | 120.1105 | 119.9372 | 29.34931 |
