# Supplementary material for: Joint Modeling of Multiple Social Networks to Elucidate Primate Social Dynamics: I. Maximum Entropy Principle and Network-Based Interactions
Source: PLoS One. 2013 Feb 28;8(2):e51903. doi: 10.1371/journal.pone.0051903 (PMC3585323; doi:10.1371/journal.pone.0051903)
Supplement: Table S12 — values of iterative joint modeling on 2009 and 2011. (DOCX) [file pone.0051903.s012.docx]

Table S12 $\hat{\lambda}_{k}$ values of iterative joint modeling on 2009 and 2011

| Year | Bivariate network | $\hat{\lambda}_{1}$ | $\hat{\lambda}_{2}$ | $\hat{\lambda}_{3}$ | $\hat{\lambda}_{4}$ |
| --- | --- | --- | --- | --- | --- |
| 2009 | groom/aggression | 2.188687 | 1.140492 | 0.5867595 | 0.3898253 |
| 2009 | groom/alliance | 2.188687 | 2.204159 | 0.1226298 | 1.477854 |
| 2009 | groom/status | 2.188687 | -2.14641 | 0.5117594 | 0.6037005 |
| 2009 | aggression/alliance | 1.140492 | 2.204159 | -0.2412303 | 0.8496505 |
| 2009 | aggression/status | 1.140492 | -2.14641 | -1.327423 | 0.3711938 |
| 2009 | alliance/status | 2.204159 | -2.14641 | -0.5217129 | 0.5039903 |
| 2011 | groom/aggression | 2.0801 | 0.8082413 | 0.6355204 | 0.5038737 |
| 2011 | groom/alliance | 2.0801 | 1.90559 | -0.2409907 | 1.480475 |
| 2011 | groom/status | 2.0801 | -0.6930715 | 0.6975685 | 0.3248612 |
| 2011 | aggression/alliance | 0.8082413 | 1.90559 | -0.3163245 | 1.025377 |
| 2011 | aggression/status | 0.8082413 | -0.6930715 | -1.397092 | 0.2621887 |
| 2011 | alliance/status | 1.90559 | -0.6930715 | -0.8840892 | 0.0980712 |
