# Supplementary material for: Joint Modeling of Multiple Social Networks to Elucidate Primate Social Dynamics: I. Maximum Entropy Principle and Network-Based Interactions
Source: PLoS One. 2013 Feb 28;8(2):e51903. doi: 10.1371/journal.pone.0051903 (PMC3585323; doi:10.1371/journal.pone.0051903)
Supplement: Appendix S1 — (DOCX) [file pone.0051903.s017.docx]

**Appendix S1:Derivation of Maximum Entropy Procedure**

To simplify notation, we let $x$ be $\left(x_{1}^{12},x_{2}^{12},x_{3}^{12},x_{4}^{12}\right)$, the four dimensional vector. We maximize the relative entropy for probability $p_{\text{m}}$ by maximizing

$$\sum_{x} p_{\text{m}}\left(x\right)log\left(\frac{p_{\text{m}}\left(x\right)}{p_{0}\left(x\right)}\right)$$

summed over all probabilities where $p_{0}$ is the null probability distribution and $p_{\text{m}}$ probability distribution with maximum entropy subject to the constraints of the data. The first constraint sets the expectation of the constraint function under the new distribution $E_{1}\left[f_{1}\left(x\right)\right]=\sum_{x} p_{1}\left(x\right)f_{1}\left(x\right)$ to be equal to the expectation of the constraint function under the empirical distribution $\widehat{E_{1}}\left[f_{1}\left(x\right)\right]=\sum_{x} f_{1}\left(x\right)\frac{d\left(x\right)}{n_{12}}$

Thus our first constraint sets:

$$\sum_{x} p_{1}\left(x\right)f_{1}\left(x\right)=\widehat{E_{1}}[f_{1}(x)]$$

The second constraint sets the the sum all probabilities to be one:

$$\sum_{x} p_{1}\left(x\right)=1$$

Therefore we can maximize the entropy using the Lagrange operation

We take the derivative of the Lagrange operation to get

$$\frac{\partial}{\partial p_{1}}L\left(p_{1},\lambda_{1},\mu\right)=\log\left(\frac{p_{1}\left(x\right)}{p_{0}\left(x\right)}\right)+1-\lambda_{1}f_{1}\left(x\right)-\mu=0$$

By solving this equation for $p_{1}$, we get

$$p_{1}\left(x\right)=p_{0}\left(x\right)exp\left(-\lambda_{1}f_{1}\left(x\right)\right)exp\left(-\mu+1\right)$$

Let $Z\left(\lambda_{1}\right)=\sum_{x} p_{0}\left(x\right)exp\left(-\lambda_{1}f_{1}\left(x\right)\right)$**,** which is called the partition function**.** Applying the constraint that all probabilities must sum to 1, we determine that

$$exp\left(-\mu+1\right)=\frac{1}{Z(\lambda_{1})}$$

Then applying the first constraint, we get

$$\sum_{x} \frac{1}{Z(\lambda_{1})}p_{0}\left(x\right)exp\left(-\lambda_{1}f_{1}\left(x\right)\right)f_{1}\left(x\right)=\widehat{E_{1}}[f_{1}(x)]$$

which is equivalent to

$$\frac{\partial}{\partial\lambda_{1}}\log Z\left(\lambda_{1}\right)=\widehat{E_{1}}[f_{1}(x)]$$

This completes the derivation previously noted in the maximum entropy procedure. In order to find $p_{1}\left(x_{1}^{12},x_{2}^{12},x_{3}^{12},x_{4}^{12}\right)=p_{1}\left(x\right)=\frac{1}{Z(\lambda_{1})}p_{0}\left(x\right)exp\left(-\lambda_{1}f_{1}\left(x\right)\right)$ we solve for $\lambda_{1}$ by the previous equation. This process can be repeated iteratively for each$f_{k}$and $p_{\text{k}}$ .
